# Model-Based Prediction of Operation Consequences When Driving a Car to Compensate for a Partially Restricted Visual Field by A-Pillars

**DOI:** 10.3389/fnhum.2021.697295

**Published:** 2021-11-04

**Authors:** Sayako Ueda, Toshihisa Sato, Takatsune Kumada

**Affiliations:** ^1^TOYOTA Collaboration Center, RIKEN Center for Brain Science, Wako, Japan; ^2^Human-Centered Mobility Research Center, National Institute of Advanced Industrial Science and Technology (AIST), Tsukuba, Japan; ^3^Graduate School of Informatics, Kyoto University, Kyoto, Japan

**Keywords:** model-based prediction, visual field restriction, visual feedback restriction, driving, visuomotor tracking task

## Abstract

The partial restriction of a driver’s visual field by the physical structure of the car (e.g., the A-pillar) can lead to unsafe situations where steering performance is degraded. Drivers require both environmental information and visual feedback regarding operation consequences. When driving with a partially restricted visual field, and thus restricted visual feedback, drivers may predict operation consequences using a previously acquired internal model of a car. To investigate this hypothesis, we conducted a tracking and driving task in which visual information was restricted to varying degrees. In the tracking task, participants tracked a moving target on a computer screen with visible and invisible cursors. In the driving task, they drove a real car with or without the ability to see the distant parts of a visual field. Consequently, we found that the decrease in tracking performance induced by visual feedback restriction predicted the decrease in steering smoothness induced by visual field restriction, suggesting that model-based prediction was used in both tasks. These findings indicate that laboratory-based task performance can be used to identify drivers with low model-based prediction ability whose driving behavior is less optimal in restricted vision scenarios, even before they obtain a driver’s license. However, further studies are required to examine the underlying neural mechanisms and to establish the generalizability of these findings to more realistic settings.

## Introduction

Although driving a car is primarily a visual task (Sivak, 1996), the physical structure of the car, such as the A-pillar between the front windscreen and the front side windows, can cause visual restrictions that have been reported to potentially contribute to road accidents, where a driver fails to notice other road users before it is too late to take appropriate action (Road Research Laboratory, 1963; Porter and Stern, 1986; Chong and Triggs, 1989; Quigley et al., 2001; Wade and Hammond, 2002; Millington et al., 2006; Reed, 2008; Marshall et al., 2012; Ekroll et al., 2021). It is reported that a driver can detect visual targets more accurately and quicker in the car with less obscuration from A-pillar than in the car with more obscuration (Quigley et al., 2001). In addition, a vast body of literature now shows that the partial obscuration of a driver’s forward field of view degrades their steering performance by preventing them from acquiring essential visual information during steering (Godthelp, 1985; Land and Horwood, 1995; Hildreth et al., 2000; Wallis et al., 2002, 2007; Frissen and Mars, 2014). Land and Horwood (1995) conducted an influential study in which they used a driving simulator to demonstrate that occlusion of the near part of the road disrupted steering accuracy, whereas occlusion of the distant part disrupted steering smoothness. Many efforts to reduce road accidents have focused on developing technologies such as ultrasonic sensors and cameras to detect visually occluded information, and then present corresponding visual, auditory, or tactile signals to the driver (e.g., Advanced Driver Assistance Systems). However, the cognitive processes that a driver recruits to steer a car with a partially restricted visual field have not been established. Identification of the cognitive processes involved in visually restricted driving is a central requirement for the effective design of driver assistance systems.

Model-based prediction of operation consequences may be employed when driving with a partially restricted visual field. In computational neuroscience fields, it has been suggested that a tool’s input-output properties are learned through practice and neurally represented as internal models that are modularly organized in the cerebellum (for a review, see Imamizu and Kawato, 2012), which allows operators to predict tool operation consequences and therefore to control tools in a feedforward manner (for a review, see Kawato, 1999). Many studies have supported this perspective. For example, using a fast-reaching task in which participants manipulated a robot arm, Dingwell et al. (2002) demonstrated that rapid and smooth behavior was reproduced better by simulating control using an acquired internal model than by relying on error-correction using visual feedback alone. Davidson and Wolpert (2004) found that after people alternately lifted two objects that were identical in physical appearance but different in weight, they could scale their grip force appropriately the first time they lifted the two objects as a combined stack. This suggests that acquired internal models can be reused to predict the consequences of new object manipulation.

One established approach for imposing model-based prediction of tool operation consequences is the restriction of visual feedback regarding the consequences of operation because the predicted operation consequences can internally compensate for restricted visual information, enabling an operator to continue to operate a tool effectively. Indeed, a substantial number of empirical studies have demonstrated that it is possible, to a certain extent, to use tools even when visual feedback regarding tool use consequences is not available (Mehta and Schaal, 2002; Hill and Raab, 2005; Ogawa and Inui, 2007; Raab et al., 2013; Ueda et al., 2019). For instance, Mehta and Schaal (2002) showed that people could continue to balance a pole in a virtual environment even when the pole was visually occluded. We previously conducted a study using functional magnetic resonance imaging (fMRI) to explore brain activity during a visuomotor tracking task in which participants were asked to track an unpredictably moving target with a visible or invisible mouse cursor (Ueda et al., 2019). The results indicated that the participants could track a moving target with an invisible mouse cursor, although the tracking performance was degraded. When the participants used the invisible cursor, the authors also found greater activation in the left cerebellum, which has been implicated in the model-based prediction of object manipulation consequences (Weiss et al., 2009; Stoodley et al., 2010, 2012; Picazio et al., 2013). This suggests that the decreased task performance associated with the restriction of visual feedback regarding tool operation consequences reflects an operator’s ability to predict tool operation consequences using an internal model.

**Figure 1:**
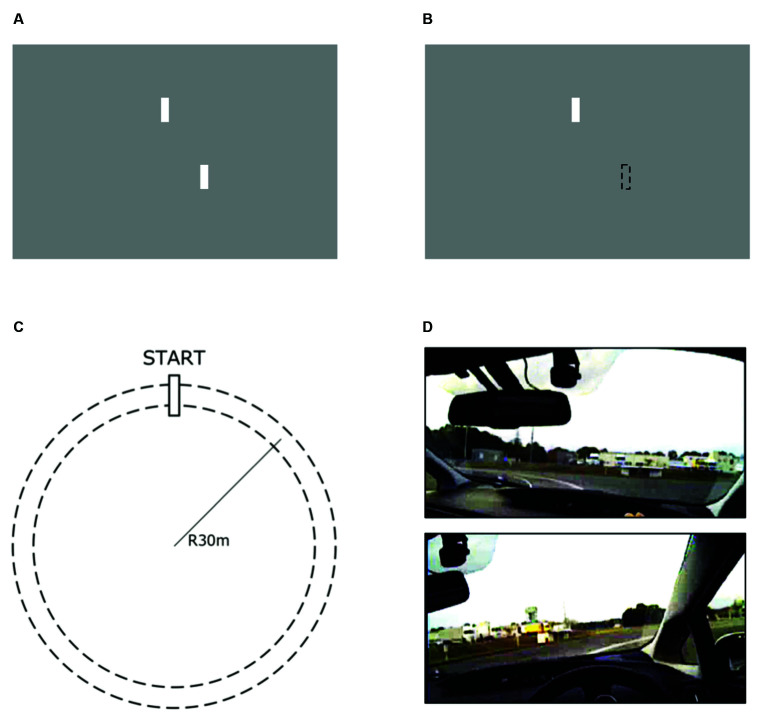
Test conditions of the tracking task **(A,B)** and driving task **(C,D)**. In the tracking task, the target and cursor are the upper and lower white bars, respectively. The target moved horizontally and unpredictably. Each participant’s task was to track the center of the target with the center of the cursor. **(A)** The visible cursor condition (VC), where the cursor was always visible, and **(B)** the invisible cursor condition (IC), where the cursor was invisible, forcing the participant to predict the position of the cursor. In the driving task, participants were instructed to drive on a circular course **(C)** while maintaining a constant speed and distance from the inner edge of the lane. In the counter-clockwise circling condition (CC: the upper figure of **D**), the distant part of the course lines was usually visible. In the clockwise circling condition (CW: the lower figure of **D**), the distant part of the course lines was occluded.

Driving a car with a partially restricted visual field may require drivers to predict the consequences of their operation using a previously acquired internal model of a car. This is because the driver’s forward field of view includes both environmental information about the road, such as information about an oncoming curve, and visual feedback regarding the consequences of their operation (for example, the driver’s entire visual field moves to the left when the car rotates to the right). If this is the case, then a driver’s model-based ability to predict the consequences of their operation should be reflected in the decrease in steering performance associated with the partial restriction of the driver’s forward field of view. The aim of this study was to investigate this possibility. To accomplish this goal, we investigated the relationship between the decrease in task performance induced by the restriction of visual feedback regarding tool operation consequences and the decrease in steering performance induced by the partial restriction of the driver’s forward field of view. We asked participants to complete two tasks. The first was a tracking task conducted in a laboratory setting, in which we measured the decrease in task performance induced by the restriction of visual feedback regarding tool operation consequences. We employed an experimental setup used in our previous study (Ueda et al., 2019): participants used a visible or invisible mouse cursor controlled by a joystick to track an unpredictably moving target on a computer screen. In this task, the participants needed to predict the cursor position using an internal model of the cursor in the invisible cursor but not the visible cursor condition. In the second task, we examined the degree to which a decrease in steering performance was associated with the partial restriction of the driver’s forward field of view in a real car driving task, where participants drove a right-hand drive car on a circular course clockwise or counter-clockwise while maintaining a constant speed and distance from the inner course line. Generally, during clockwise driving with a right-hand drive car, the right front pillar partially occludes the distant part of the course lines more than during counter-clockwise driving (see Figure 1D). In this task, therefore, the participants were expected to drive the car more predictably when driving clockwise vs. counter-clockwise. Using contrasting conditions in terms of the level of available visual information enabled us to clarify the degree to which task performance was degraded and remove components related to individual differences outside the study scope (i.e., model-based prediction of the consequences of participants’ operation), such as motor control abilities or task-specific skills (i.e., tracking skill and driving skill). We hypothesized that if the ability to predict the consequences of participants’ operation using an internal model is helpful in not only the invisible-cursor tracking task but also the partially-restricted-view circular driving task (i.e., clockwise driving), then the change in task performance in the tracking and driving tasks would be correlated.

## Methods

### Participants

Twenty experimentally naïve adults (12 females; eight males) aged 20–50 years (*M* = 36.5 years; *SD* = 10.2 years) participated in this study and received payment for their participation. All participants had a normal or corrected-to-normal vision and were right-handed according to the Edinburgh Handedness Inventory (Oldfield, 1971). They reported that they drove a car on a daily basis. Written informed consent was obtained in accordance with a protocol approved by the RIKEN Research Ethics Committee [Wako3 28-17(4)].

### Apparatus, Setup, and Procedure

#### Tracking Task

The experiment was conducted in a room with natural light. Participants were seated at a distance of 60 cm, fixed by a forehead and chin rest, from a 27-inch computer screen (2,560 × 1,440 pixels, 53.1° × 31.4° in visual angle). They tracked a moving target on the screen using a cursor, which was controlled by a joystick (Logitech Extreme 3D Pro) held with both hands. Both the target and cursor were identical small white bars (5 × 50 pixels), each presented on a gray background. A target bar was located on the upper third of the screen, and the cursor bar was located 30 pixels below the target (see Figure 1A). The target bar moved horizontally (i.e., the x-coordinate was variable and y-coordinate was fixed). Its trajectory was generated using the sum of two sinusoidal waves so that participants could not predict target movements. The angle of the joystick from the original position corresponded linearly to the horizontal distance of the cursor bar from the screen center. The position of the cursor bar was recorded at 30 Hz. Presentation of stimuli and recording of participant tracking trajectories were controlled by a computer using the MATLAB platform with the Psychophysics Toolbox (Brainard, 1997; Pelli, 1997).

Participants were asked to track the target bar with either a visible cursor bar (the visible cursor condition; named VC, Figure 1A) or an invisible cursor bar (the invisible cursor condition; named IC, Figure 1B) while minimizing the horizontal distance between the positions of the target and cursor bars. All participants were given a practice session composed of five trials in the VC condition to familiarize them with the tracking task. In the following test session, all participants performed five trials in the VC condition first and then performed five trials in the IC condition. For the test session, we prepared five different target movements that were different from the ones prepared for the practice session, and each movement was used twice, once in the VC condition and once in the IC condition. The order of target movements was randomized in each condition. One trial lasted 16.6 s and was divided into three epochs: the first epoch (5 s), second epoch (8.3 s), and third epoch (3.3 s). In the VC condition, the target and cursor were visible throughout all epochs. In the IC condition, the cursor was not visible only during the second epoch. After finishing one trial, participants could proceed to the next trial at their own pace by pushing a button on the joystick.

#### Driving Task

The experiment was conducted in a wide, flat, and asphalt-paved field during the daytime under good weather conditions (i.e., no rain). Participants drove a right-hand-drive passenger vehicle (Prius ZVW30, Toyota) that was equipped with various sensors to record the vehicle’s driving states (e.g., geographical position) and to measure the participant’s operation behavior (e.g., steering angle). The circular course had a radius of 30 m and consisted of a lane 2.8 m wide (Figure 1C). Participants were asked to execute smooth circular maneuvers for ten laps in either the counter-clockwise direction (the counter-clockwise circling condition; referred to as CC) or the clockwise direction (the clockwise circling condition; CW) while trying to maintain a speed of 40 ± 3 km/h and keeping a constant distance from the inner course line. However, in the CW condition, the right front pillar occludes the distant part of the course lines (Figure 1D). Participants started circling by accelerating their vehicle to the specified speed in the first lap after the starting point, and then maintained this speed from the second to the ninth lap. Finally, they decelerated during the final lap and then stopped their vehicle. The order of both circling conditions was counterbalanced across participants.

The geographical position of the vehicle was obtained using an RTK-GPS (Real Time Kinematic—Global Positioning System) sensor (NovAtel, GPS-700 Series) and its data were sampled at 10 Hz. Steering angle data were sampled at 30 Hz. These data were recorded by a laptop computer onto mobile hard disks. To encourage participants to maintain natural driving behavior, all equipment was fixed inside the trunk of the vehicle where the instruments could not be seen.

### Data Analysis

#### Tracking Performance

Tracking performance was evaluated using* tracking error*, defined as the root mean square of the position error (RMSE), the distance between the centers of the target and cursor, which is a common performance index for visuomotor tracking tasks in previous studies (Hill and Raab, 2005; Raab et al., 2013; Ueda et al., 2019). A larger tracking error implies less accurate tracking. For the IC condition, only the data that were collected while the cursor was not visible (i.e., the second epoch) were analyzed. Similarly, for the VC condition, only the data that were collected during the second epoch were analyzed, to equalize the data quality between the VC and IC conditions and to exclude noisy data at the start and end of movements.

The tracking error metric in the practice session could be assumed to be the highest in trial 1, then decrease with repetition of trials, and finally reach a plateau when participants were sufficiently trained to acquire an internal model of the cursor. To assess this trend, first, the data in trials 1–5 were subjected to a one-way repeated measures analysis of variance (ANOVA). When a significant effect of the trial was noted, another one-way repeated ANOVA was performed after removal of the data of the smallest trial number (for example, trial 1). This step was repeated until no significant effect of the trial was discovered at which point it could be judged that the tracking error metric had reached a plateau.

The tracking error metric in the test session was subjected to a two-way repeated ANOVA with trial (i.e., trial 1, 2, 3, 4, and 5) and condition of visual information restriction (i.e., VC and IC conditions) as factors to examine whether visual information restriction disrupts tracking performance, as reported in previous studies, and to confirm that there was no significant difference between trials after acquiring the internal model of the cursor.

#### Driving Performance

Driving performance was evaluated using two metrics: *accuracy* and *smoothness*. The accuracy metric is defined as the standard deviation of lateral position (SDLP) of the vehicle, which is the standard deviation of the difference between the center of the path and the center of the vehicle in the driving task. In contrast, the smoothness metric is measured as the cumulative root squared jerk (CRSJ), which is the second derivative of the steering wheel velocity. Steering accuracy and smoothness metrics are common performance indices for the lane-keeping task in previous studies (Land and Horwood, 1995; Frissen and Mars, 2014), and it has been demonstrated that restricting the view of the distant part of the road compromises the steering smoothness metric but not the accuracy metric. For both metrics, lower values represent better performance. To exclude noisy data at the start and end of movements, only the data from the 2nd to 9th laps were analyzed.

The steering accuracy and smoothness metrics in the test session were separately subjected to a two-way repeated ANOVA with lap and condition of visual information restriction (i.e., CC and CW conditions) as factors to examine whether visual information restriction disrupts driving performance, as reported in previous studies, and to confirm that there was no significant difference between laps in the test session.

#### Tracking vs. Driving Performance

Finally, the reduced performance caused by visual information restriction was calculated by normalizing (i.e., dividing) the performance obtained under the restricted visual information condition (i.e., IC and CW) by the performance obtained under the unrestricted visual information condition (i.e., VC and CC, respectively). Regarding these metrics, as their values approach 1, the effect of visual information restriction on each performance metric is less. It should be noted that the normalization procedure enabled the removal of components related to individual differences outside the study scope (i.e., the reduced performance caused by visual information restriction), such as motor control abilities or each task performance. Therefore, it is possible that the values of the normalized performance metrics were high even when the values of the performance metrics were low under the unrestricted visual information condition.

The reduced performance metrics were then separately subjected to a Pearson’s correlation analysis between tasks (i.e., the tracking task vs. driving task) to examine whether the effect of visual information restriction on tracking performance is related to the effect of visual information restriction on driving performance. In addition, the reduced performance metrics were also separately subjected to a simple linear regression analysis to assess whether the effect of visual information restriction on tracking performance predicted the effect of visual information restriction on driving performance. A significance threshold was set at *p* < 0.05 for all tests.

## Results

The data of one female participant was excluded from analyses because she failed to stay awake during the two test trials of the tracking task. Therefore, the following analyses were performed on data from the remaining 19 participants.

### Tracking Performance

**Figure 2:**
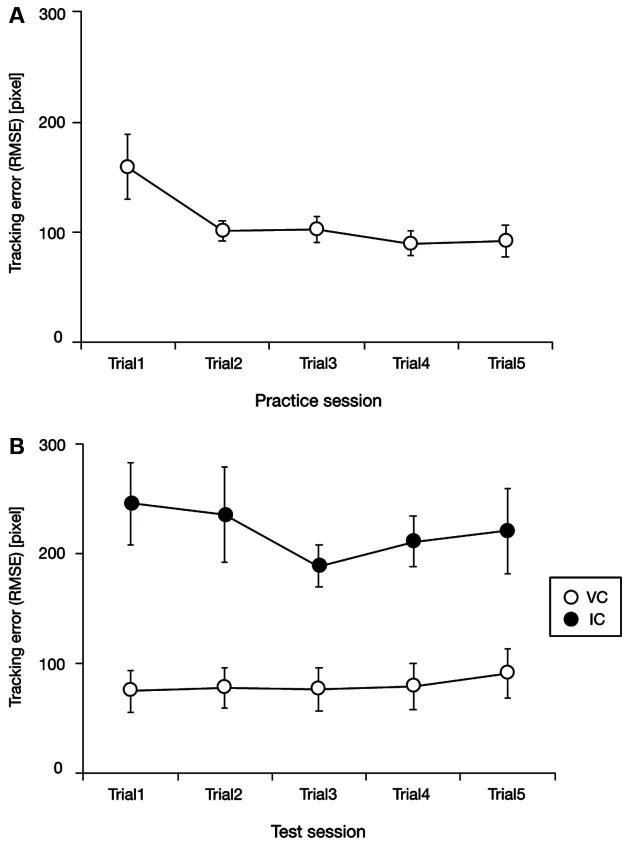
Tracking performance as a function of trial in the practice session **(A)**, and tracking performance as a function of trial for each task condition in the test session **(B)**. Tracking performance tended to decrease and then plateau during the practice session. In the test session, tracking performance seems to be worse in the invisible cursor condition (IC) than in the visible cursor condition (VC). RMSE denotes the root mean square of the position error. Error bars represent 95% confidence intervals.

Performance changes during the practice session are shown in Figure 2A. The tracking error metric tends to decrease and reach a plateau during the first two trials. To assess this trend statistically, we performed a one-way repeated ANOVA using the data of trials 1–5, which yielded a significant effect of trial (*F*_(4,72)_ = 6.23, *p* < 0.01, $\eta_{p}^{2}$ = 0.26). We then performed a one-way repeated ANOVA using the data of trials 2–5, which yielded no significant effect of the trial (*F*_(3, 54)_ = 0.99, *p* = 0.40, $\eta_{p}^{2}$ = 0.05). These results suggest that participants were sufficiently trained during the first two trials in the practice session.

**Figure 3:**
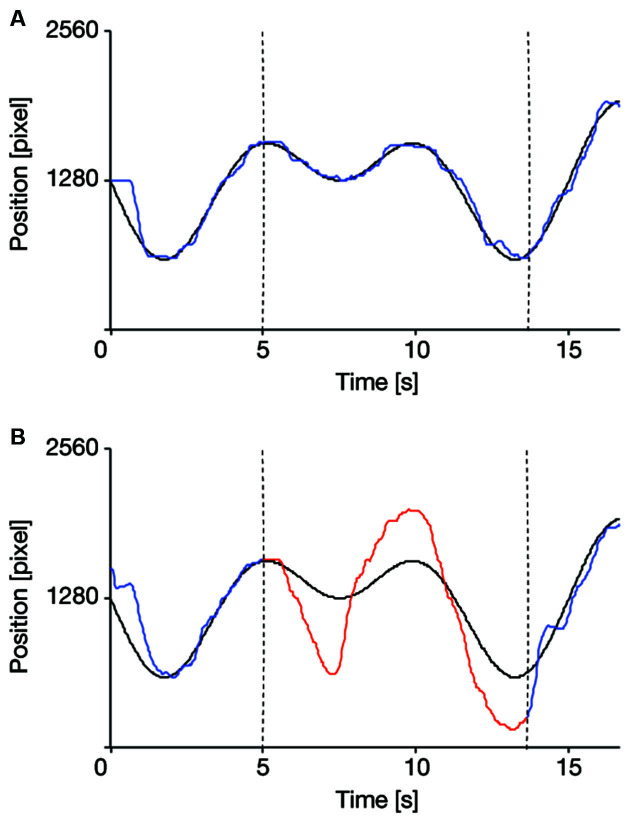
Examples of the target and cursor movements in the visible cursor condition (VC: **A**, blue line) and the invisible cursor condition (IC: **B**, red line). The values of 0 and 2,560 on the vertical axis respectively indicate the left and right sides of the display. Dotted black lines represent epoch switching points. Black curved lines are the target trajectories, and the colored lines are the typical cursor movements of one of the participants. Note that in the IC condition **(B)** for the first and last epochs of each trial, visual feedback for cursor position was provided, as indicated by blue lines. These periods in the IC condition were excluded from data analysis.

Figure 3 illustrates examples of the target and cursor movements in the VC and IC conditions during the test session. The cursor movement seems to follow the target trajectory accurately in the VC condition (Figure 3A). In the IC condition (Figure 3B), a typical pattern of cursor movement repeatedly reported in previous tracking studies (Hill and Raab, 2005; Ogawa and Inui, 2007; Raab et al., 2013; Ueda et al., 2019) can be seen; that is, the cursor keeps following the target trajectory even during the invisible phase, although the cursor position is overpredicted. In fact, no participants dropped out of tracking even after the cursor was made invisible, suggesting that they successfully acquired the internal model of the cursor during the practice session and therefore used it to predict cursor position when the cursor was invisible in the IC condition of the test session.

Test session performances are shown in Figure 2B. The tracking error metric in the IC condition seems to be worse than that in the VC condition. To assess this trend, we performed a two-way repeated ANOVA with trial and condition of visual information restriction as factors. As expected, the results revealed a significant effect of condition of visual information restriction (*F*_(1, 18)_ = 37.78, *p* < 0.01, $\eta_{p}^{2}$ = 0.68), no significant effect of trial (*F*_(4, 72)_ = 1.09, *p* = 0.37, $\eta_{p}^{2}$ = 0.06) and no significant interaction between trial and condition of visual information restriction (*F*_(4, 72)_ = 1.28, *p* = 0.29, $\eta_{p}^{2}$ = 0.07). These findings suggest that tracking performance was degraded by the visual feedback restriction.

### Driving Performance

**Figure 4:**
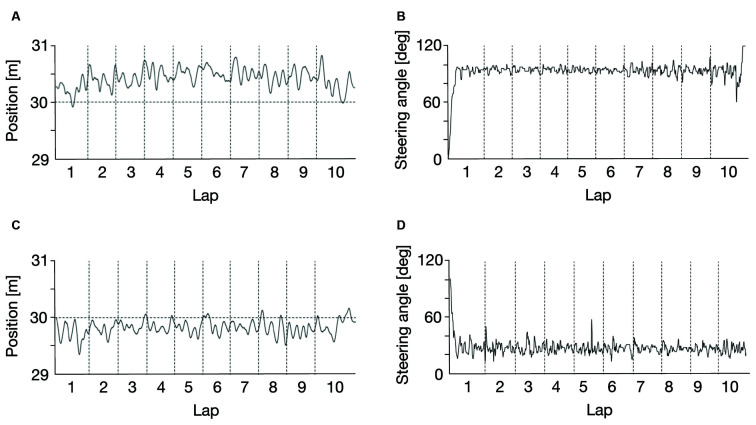
Examples of vehicle position in the counter-clockwise circling condition (CC: **A**) and the clockwise circling condition (CW: **C**), and steering operation in the CC condition **(B)** and the CW condition **(D)** in the test session. Dotted horizontal black lines at 30 m in **(A,C)** indicate the center of the circular path, and black curved lines are the typical car movements **(A,C)** and steering operation **(B,D)** of one of the participants. The data of the first and last laps were excluded from data analysis.

Figure 4 illustrates examples of the vehicle position (CC: Figure 4A, CW: Figure 4C) and the steering operation (CC: Figure 4B, CW: Figure 4D) during the test session. In both the CW and CC conditions, the vehicle position appears to be comparably stable, even when the distant part of the visual field is naturally restricted by elements of the car frame in the CW condition. In contrast, the steering operation seems to be jerkier during laps in the CC condition.

**Figure 5:**
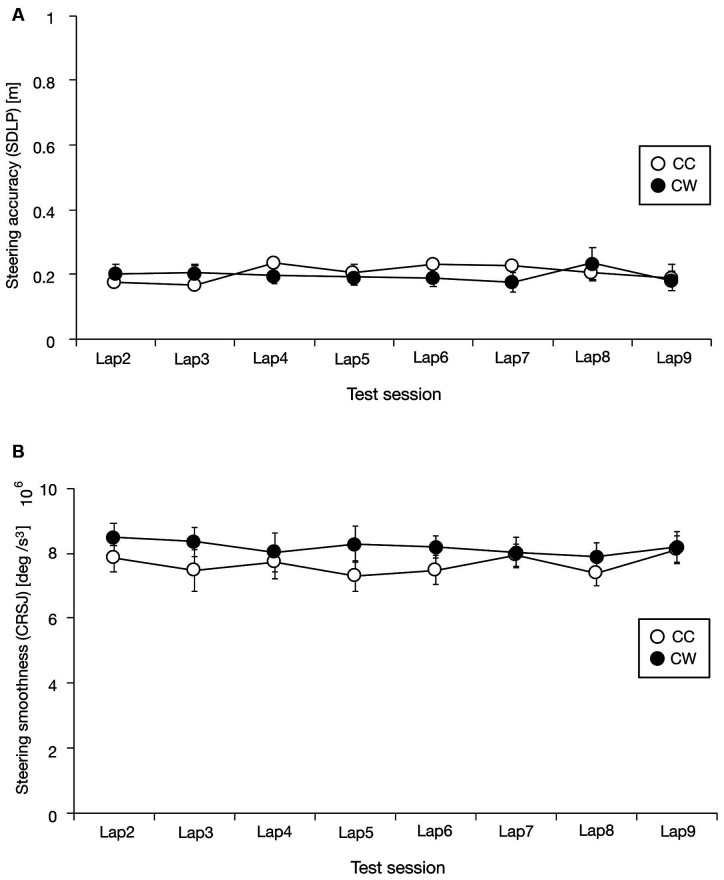
Driving performance as a function of a lap in the test session [steering accuracy: **(A)**, steering smoothness: **(B)**]. Steering accuracy seems to be comparable in both the counter-clockwise circling condition (CC) and the clockwise circling condition (CW) whereas steering smoothness seems to be worse in the CW condition than in the CC condition. SDLP and CRSJ denote the standard deviation of lateral position and the cumulative root squared jerk, respectively. Error bars represent 95% confidence intervals.

The steering accuracy and smoothness metrics for the test session are shown in Figure 5A (SDLP for the steering accuracy metric) and Figure 5B (CRSJ for the steering smoothness metric). As mentioned above, the steering accuracy metrics for both the CC and CW conditions seem to be comparable, but the steering smoothness metric seems to be larger in the CW condition. To assess these trends statistically, we performed a two-way repeated ANOVA test with lap and condition of visual information restriction as factors, separately for the steering accuracy and smoothness metrics. As expected, the results showed that the effect of condition of visual information restriction was not significant for the steering accuracy metric (*F*_(1, 18)_ = 0.22, *p* = 0.64, $\eta_{p}^{2}$ = 0.01) but was significant for the steering smoothness metric (*F*_(1, 18)_ = 4.69, *p* = 0.04, $\eta_{p}^{2}$ = 0.21), the effect of lap was not significant for either the steering accuracy metric (*F*_(7, 126)_ = 0.84, *p* = 0.56, $\eta_{p}^{2}$ = 0.04) or the steering smoothness metric (*F*_(7, 126)_ = 0.64, *p* = 0.72, $\eta_{p}^{2}$ = 0.03), and the interaction between lap and condition of visual information restriction was not significant for either the steering accuracy metric (*F*_(7, 126)_ = 1.17, *p* = 0.33, $\eta_{p}^{2}$ = 0.06) or the steering smoothness metric (*F*_(7, 126)_ = 0.73, *p* = 0.65, $\eta_{p}^{2}$ = 0.04). These results suggest that the steering smoothness was degraded by the restriction of the distant part of the visual field, but accuracy was not affected.

### Tracking vs. Driving Performance

**Figure 6:**
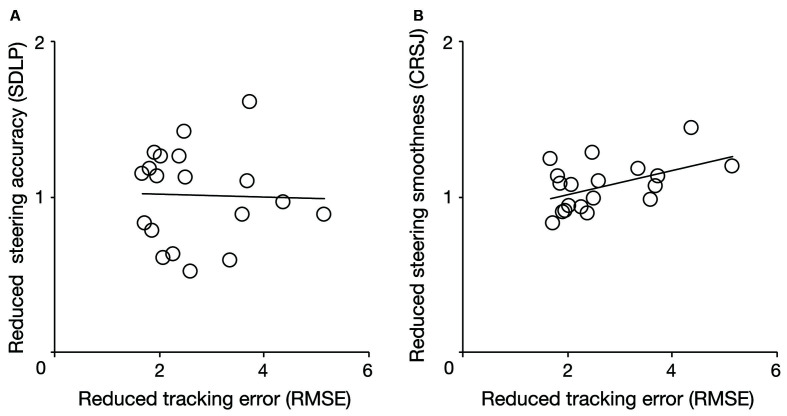
Correlation of the reduced tracking performance with the reduced driving performance [steering accuracy: **(A)**, steering smoothness: **(B)**]. The reduced tracking performance was calculated by normalizing the values of the indices in the invisible cursor condition (IC) by those in the visible cursor condition (VC), and the reduced driving performance was calculated by normalizing the values of the indices in the clockwise circling condition (CW) by those in the counter-clockwise circling condition (CC). RMSE, SDLP, and CRSJ denote the root mean square of the position error, the standard deviation of lateral position, and the cumulative root squared jerk, respectively.

Figure 6 shows scatter diagrams of the reduced tracking performance, which was calculated by normalizing the values of the IC condition by those in the VC condition, and the reduced driving performance, which was calculated by normalizing the values of the indices in the CW condition by those in the CC condition (Figure 6A; SDLP, Figure 6B; CRSJ). There is no significant correlation between the reduced tracking error and steering accuracy metrics (*r* = −0.03, *p* = 0.90) and the regression line was also not significant (*F*_(1, 17)_ = 0.02, *p* = 0.91, adjusted* R^2^* = −0.06). However, the reduced tracking error metric was significantly correlated with the reduced steering smoothness metric (*r* = 0.49, *p* = 0.03) and the regression line was significant (*F*_(1, 17)_ = 5.42, *p* = 0.03, adjusted *R^2^* = 0.20). These results indicate that the decrease in an individual’s tracking performance induced by visual feedback restriction can predict their steering smoothness but not the decrease in accuracy induced by the restriction of a distant part of participants’ visual field.

## Discussion

Accumulating evidence suggests that the degree of restriction of a driver’s forward field of view by the physical structure of a car is a contributory factor in the degradation of the driver’s steering performance. When driving a car with a partially restricted visual field, the driver may recruit model-based prediction of operation consequences, which has been shown to be imposed in situations where visual feedback regarding operation consequences is restricted. This is because a driver’s visual field includes not only environmental information but also visual feedback regarding the operation consequences. However, to date, no studies have directly investigated this possibility. Therefore, in the present study, we conducted a laboratory tracking task and a real car driving task with different levels of restriction of visual information. We examined the relationship between the decrease in tracking performance induced by visual feedback restriction and the decrease in driving performance induced by the partial restriction of the distant part of a visual field. We hypothesized that if the participants successfully used model-based prediction of their operation consequences in both the tracking task with restricted visual feedback and the partially-restricted-view driving, then the change in task performance in the tracking and driving tasks would be correlated.

We found that after sufficient practice, the participants could track a moving target with the invisible cursor, although the performance was degraded compared with that in the visible cursor condition. This indicates that model-based prediction of the cursor position enabled the participants to continue to operate the cursor without visual feedback regarding operation consequences, although it was difficult to track the target accurately. This is a robust replication of previous findings (Hill and Raab, 2005; Ogawa and Inui, 2007; Raab et al., 2013; Ueda et al., 2019). In addition, during the driving task, the participants were able to maintain accurate circular driving even when the distant part of the visual field was naturally restricted by elements of the car frame, although steering smoothness was degraded. This indicates that visual information about the distant part of the road is critical for smooth steering but not for accurate lane-keeping, which is also consistent with previous findings (Land and Horwood, 1995; Frissen and Mars, 2014). More importantly, we found that the tracking and driving performances were correlated, and tracking performance partially predicted driving performance. Specifically, the decrease in tracking performance caused by the restriction of visual feedback in the tracking task could predict the decrease in steering smoothness induced by the obscuration of the distant part of the visual field in the driving task. This suggests that individuals who cannot accurately predict the cursor position using an internal model of a cursor are susceptible to altered driving ability induced by obscuration of the visual field. Therefore, it appears that model-based predictions regarding operation consequences occurred not only in the laboratory-based tracking task with restricted visual feedback but also in the real car driving task with the partially restricted visual field. This constitutes an important and novel finding, as no previous studies have successfully identified an ability used to cope with a partially restricted visual field when driving a car. Accordingly, this information may be helpful in the development of effective driver assistance systems.

In contrast to steering smoothness, the decrease in tracking performance induced by restricted visual feedback was not correlated with the decrease in steering accuracy induced by occlusion of the visual field during the driving task. This is in line with a widely accepted theoretical framework proposed by Donges (1978), which holds that steering accuracy and smoothness are determined by two independent control processes. The first is an anticipatory process that uses distant visual information to generate smooth steering behavior, and the second is a compensatory process that uses proximal visual information to enable accurate steering. In the present study, the distant part of the road was always occluded in the clockwise condition. Assuming that the participants could not properly perform anticipatory control in this condition, the decrease in steering smoothness may reflect decreased engagement of anticipatory control. Therefore, we speculate that drivers who were able to compensate for the occluded distant part of the visual field using model-based prediction of steering operation consequences retained anticipatory control. As the proximal part of the visual information was always visible in the present study, the participants could use it to perform compensatory control comparably well in both conditions. Therefore, we speculate that the decrease in tracking performance induced by the restricted visual feedback was correlated with the decrease in steering accuracy induced by the occluded proximal part of the visual field. Moreover, the decrease in steering accuracy caused by the occluded proximal part of the visual field should reflect a lower engagement of compensatory control. Confirmation of these speculations is a direction for future research. Taken together, the present results are consistent with the two-level theoretical framework of steering control, and suggest that model-based prediction of steering operation consequences can compensate for occlusion of the distant part of a visual field.

Our data may be useful in the development of new practical screening tools that could predictively identify drivers who cannot predict the consequences of their operation and therefore easily lose steering smoothness as a result of partially restricted visual information during driving, even before they obtain a driver’s license. In real-world traffic environments, the loss of steering smoothness based on the driver’s low level of ability to predict the consequences of their operation may contribute to creating an unsafe driving situation. For example, when avoiding an obstacle on the road while driving with occlusion of the distant part of the visual field, the position after obstacle avoidance was not significantly different between drivers with high and low ability to predict the consequences of their operation because steering accuracy was preserved. However, the driving path to the position and the steering state could be disrupted in drivers with a low ability to predict the consequences of their operation because of the loss of steering smoothness; this could potentially result in delayed responses to subsequent traffic events, such as avoiding pedestrians. In the past decade, many screening tools for replacing on-road assessments have been developed and extensively studied. For example, in a hazard perception test created by Wetton et al. (2010), users view video clips of traffic fields filmed from the driver’s point-of-view. Users are asked to identify potential traffic hazards by pressing the relevant area of a touch-screen whenever they identify a potential incident. This test was developed to measure how quickly drivers could anticipate hazards by incorporating detection, trajectory, and hazard classification judgments. Furthermore, the multi-disciplinary driving assessment battery by Wood et al. (2008) requires participants to attend two laboratory-based sessions that include assessments of vision, cognitive function, sensorimotor performance, and balance. It was created to predict safe and unsafe performance during on-road driving assessments. However, to date, there are no screening tools that assess a driver’s ability to predict the consequences of their operation using an internal model. Drivers who have low model-based prediction ability could perform less optimally when visual information is restricted, even when they show a high level of driving skill in a situation without visual restrictions. Therefore, our results may represent the origin of a new approach to predicting which drivers will perform less optimally in a situation-dependent manner, rather than predicting drivers whose driving behavior is always less optimal.

The conclusion that model-based predictions regarding operation consequences occurred not only in the laboratory-based tracking task but also in the real car driving task suggests that there is a common processing component regarding the model-based prediction of operation consequences across the neural mechanisms that underlie a wide range of different tool operation situations. This possibility is in accord with previous findings that propose the existence of hierarchical structures of the functions involved in tool operation. For example, Imamizu et al. (2007) showed that people could switch between acquired internal models when using a cursor controlled by a finger to point to a target on a computer screen immediately after the relationship between the finger direction and cursor movement had been altered. As mentioned in the Introduction, Davidson and Wolpert (2004) demonstrated that people could use previously acquired internal models to predict the consequences of new object manipulation. These results indicate that there could be a higher-order function for managing various internal models. However, in the current experiments, it was unclear whether the common component was only related to model-based prediction of operation consequences. It is also possible that tool-specific components exist, which are related to the model-based prediction of operation consequences to cope with differences across a wide range of different tool operation situations. For example, in the current study, the tracking task only required participants to track the moving target using the invisible cursor in a fixed environment, and participants simply predicted the cursor position. However, in the driving task, the surrounding environments changed while participants drove a car, and they were also provided with not just visual feedback but also other types of sensory feedback, such as auditory and vestibular feedback. In such a situation, the predictions that participants made while driving a car with a partially restricted visual field would be more complex than those in the tracking task. If not only common but also tool-specific processing components are related to the model-based prediction of operation consequences, the cerebellar areas that we previously found to be related to the model-based prediction of operation consequences (Ueda et al., 2019) may be divided into two areas: one for the tool-common model-based prediction of operation consequences and the other for the tool-specific model-based prediction of operation consequences. However, this speculation should be confirmed directly in future studies using fMRI.

Finally, several limitations of this study should be noted. First, we were unable to rule out the possibility that the decrease in steering smoothness might not have been induced only by obscuring of the distant part of the visual field in the driving task because other differences existed between the CC and CW conditions, including the driver’s position in the car, which could have potentially caused a difference in the forces felt by the driver during circular driving. Further studies with more sophisticated experimental designs and methods will be required to eliminate this possibility. Second, the small sample size restricts the generalizability of the current finding regarding the correlation between tracking and driving performance. Replication with a larger sample size is needed to improve the generalizability of our results. The third limiting factor relates to the experimental setting of the real car driving task. In the current study, we instructed the participants to drive while maintaining a specific speed (40 ± 3 km/h) and keeping a constant distance from the inner edge of the lane. Thus, the participants might not have performed their usual steering operations because the experimental setting was unlike a daily real-world driving situation. To clarify the relationship between the model-based prediction of tool operation consequence and driving performance in real-world traffic environments with a partially restricted visual field, the driving task should be conducted in more realistic traffic situations.

## Conclusions

Our findings represent the first behavioral evidence that drivers recruit model-based prediction of operation consequences not only in a laboratory-based tracking task with restricted visual feedback but also in a real car driving task with a partially restricted visual field. This indicates that simple laboratory-based task performance could be used to predict drivers whose driving behavior is less optimal in situations with partially restricted visual fields on the basis of their diminished ability to predict operation consequences using an internal model. Further studies are required to identify the neural substrates that underlie the link between the model-based prediction of operation consequences in the laboratory-based tracking task and the real car driving task and to generalize these findings to more realistic daily driving settings.

## Data Availability Statement

The raw data supporting the conclusions of this article will be made available by the authors, without undue reservation.

## Ethics Statement

The studies involving human participants were reviewed and approved by the RIKEN Research Ethics Committee. The patients/participants provided their written informed consent to participate in this study.

## Author Contributions

SU developed the study concept. All authors contributed to the study design. Data collection was performed by SU and TS. Data analysis and drafting the manuscript were performed by SU. TK helped drafting the manuscript. All authors contributed to the article and approved the submitted version.

## Conflict of Interest

The authors declare that this study received funding from Toyota Motor Corporation. The funder was not involved in the study design, collection, analysis, interpretation of data, the writing of this article or the decision to submit it for publication.

## Publisher’s Note

All claims expressed in this article are solely those of the authors and do not necessarily represent those of their affiliated organizations, or those of the publisher, the editors and the reviewers. Any product that may be evaluated in this article, or claim that may be made by its manufacturer, is not guaranteed or endorsed by the publisher.
